# Sex Bias in Asthma Prevalence and Pathogenesis

**DOI:** 10.3389/fimmu.2018.02997

**Published:** 2018-12-18

**Authors:** Ruchi Shah, Dawn C. Newcomb

**Affiliations:** ^1^Department of Medicine, Vanderbilt University Medical Center, Nashville, TN, United States; ^2^Department of Pathology, Microbiology and Immunology, Vanderbilt University, Nashville, TN, United States

**Keywords:** asthma, allergic disease, sex hormones, puberty, pregnancy, menopause

## Abstract

Sex-related differences in asthma prevalence are well established and change through the reproductive phases of life. As children, boys have increased prevalence of asthma compared to girls. However, as adults, women have increased prevalence of asthma compared to men. Many factors, including genetics, environment, immunological responses, and sex hormones, affect the sex disparity associated with the development and control of asthma and other allergic diseases. Fluctuations of hormones during puberty, menstruation, pregnancy, and menopause, alter asthma symptoms and severity. In this article, we review clinical and epidemiological studies that examined the sex disparity in asthma and other allergic diseases as well as the role of sex hormones on asthma pathogenesis.

## Introduction

There is a sexual dimorphism in asthma and allergic disease that changes through life. Among children, boys have an increased prevalence of asthma and allergic disease compared to girls. Interestingly, around puberty the frequency of asthma and allergic disease starts to change from being higher in males to higher in females. By adulthood, the prevalence of asthma and allergic disease is increased in women compared to men (1). This change in prevalence around puberty suggests sex hormones and other factors alter pathways important in asthma pathogenesis and allergic disease.

**Figure 1:**
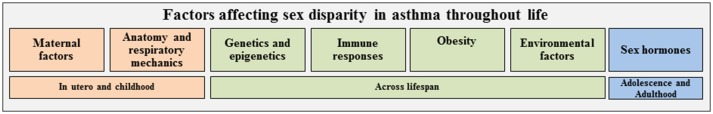
Multiple factors are associated with asthma and may contribute to the sex disparity seen in asthma throughout the life span. These factors may independently or jointly be associated with asthma or regulate each other (e.g., genetics may impact immune response and/or obesity). Factors are color-coded based on importance in asthma at various phases of life: orange, *in utero* and childhood; green, throughout life; blue, adolescence and adulthood.

Asthma is a heterogeneous disease characterized by episodes of airway narrowing or hyper responsiveness, obstruction, inflammation and mucous production. Asthma clinically presents as wheezing, coughing, chest tightness and shortness of breath (2, 3), and different inflammatory pathways drive the airway inflammation and hyperresponsiveness associated with asthma. Patients with allergic asthma have increased eosinophils in the airway or bronchoalveolar lavage fluid (BAL) caused by increased type 2 inflammation. Type 2 inflammation is characterized by increased production of interleukin (IL)-4, IL-5, and IL-13, increased IgE antibody production, and mast cell or basophil degranulation (4–6). However, some patients with more severe phenotypes of asthma have increased neutrophils in the airway and BAL fluid that is driven by IFN-γ or IL-17A-mediated pathways (4, 5). Patients with increased airway neutrophils may or may not have increased airway eosinophils as well. Multi-variate cluster analyses on adults with asthma and healthy controls determined a female predominance in two clusters: (1) less atopic, less corticosteroid responsive patients and (2) late-onset, more severe phenotypes of asthma in obese patients (4, 7). As summarized in Figure 1, studies also highlighted the many different factors, including genetics and epigenetics, environment, respiratory mechanics, immunological responses, sex hormones and obesity, regulated asthma pathophysiology and the various endotypes seen in asthma throughout

the lifespan. Therefore, understanding how sex hormones and other factors regulate asthma pathogenesis is important since asthma affects approximately 235 million people with a global annual health care burden in 2007 of approximately $56 billion for medical costs, lost school and work days and early deaths (8). In this review, we primarily focused on the sex differences and sex hormone regulation in asthma pathogenesis in the clinical and epidemiological literature organized by maternal factors, childhood asthma, and asthma after puberty (Table 1). At the end of the review, there will be a brief overview of the sex disparity in other allergic diseases.

**Table 1:** Summary of clinical findings for gender disparities in asthma during various reproductive phases of life.

**Authors**	**Methods**	**Demographics and number of subjects**	**Conclusions**
**ASTHMA AND PUBERTY**			
Fu et al. (9)	- Longitudinal study for asthma symptom diary scores (CAMP data) - Tanner stage scores	- 5 to 17 yr olds - 418 subjects - 564,518 records	- 5–6 yrs: asthma severity: M > F - 7–9 yrs: no sex difference in severity - 10–17 yrs: asthma severity: F > M
Wijga et al. (10)	- Longitudinal study, questionnaire based data collected during pregnancy, 3 months, 1 yr and yearly thereafter (PIAMA data)	- 3,308 children followed birth-8 yrs	- 0–3 yrs: incidence of asthmatic wheeze: M >F - 4–7yrs: no significant difference - 8 yrs: atopy prevalence: M > F
Vink et al. (11)	- Longitudinal study, questionnaire based data	- 2,230 dutch adolescents	- At mean age 11.1: asthma prevalence: boys = girls - At mean age 16.3: asthma prevalence: F > M
Chen et al. (1)	- Retrospective study on canadian hospital records over 3 yrs	- 288,977 asthma-related records - 204,304 asthma patients	- 3 yrs cumulative incidence of asthma hospitalization: boys> girls; reversed in adults - 25–34 yrs of age, incidence ratio for asthma hospitalization 2.8 F:M
Schatz el al. (12)	- Retrospective study on computerized data used to identify and analyze asthmatic patients with regard to asthma related HCU	- 60,694 subjects (2–64 yrs)	- Ages 2–13 yrs: asthma HCU and severity: M > F - Ages 14–22: asthma HCU and severity: F > M - Ages 23–64: asthma HCU and severity: F > M
Nicolai et al. (13)	- Longitudinal study, asthmatic and control patients recruited at age 10, re-evaluated at age 14 and 20	- 274 asthmatics and 1,000 healthy controls (ages 10–20)	- At 20 yrs, 24.5% still had symptoms (M > F) and 4.8% had developed asthma (F > M)
**ASTHMA AND THE MENSTRUAL CYCLE**			
Shames et al. (14)	- Daily asthma symptoms, medication use, PEFRs, spirometry and methacholine challenges longitudinally over 6 menstrual cycles	- 32 asthmatic women	- 28.2% of subjects reported PMA. - Women with PMA had increased perimenstrual use inhaled SABA and decreased morning PEFRs.
Pauli et al. (15)	- Daily asthma symptom diaries, PEFRs, spirometry and methacholine challenges longitudinally during 3–4 menstrual cycles	- 11 asthmatic women and 29 healthy controls	- AM PEFRs and asthma symptoms from follicular to luteal phase: asthmatics > controls - No significant changes in spirometry and airway reactivity
Rao et al. (16)	- Questionnaire based data (SARP), •[-]Inflammatory markers, spirometry	- 756 women; 483 self-reported PMA	- Use of oral corticosteroid bursts and HCU: women with self-reported PMA > women without PMA
Brenner et al. (17)	- ED interview •[-]Medical record review •[-]Visits classified by menstrual phase	- 792 asthmatic women (18–54 yrs)	- Acute asthma exacerbations do not markedly increase during perimenstrual phase -Preovulatory + perimenstrual phases may have adverse impact
Zimmermanet al. (18)	- ED interview •[-]Medical record review •[-]Visits classified by menstrual phase	- 288 asthmatic women	- Menstrual phase at time of ED visit: 33% preovulatory, 26% periovulatory, 20% postovulatory, and 21% perimenstrual
Eliasson et al. (19)	- Survey based data	- 57 asthmatic women	- 33% had increased pre or perimenstrual pulmonary symptoms
Gibbs et al. (20)	- Questionnaires and twice daily PEFRs	- 126 asthmatic women (14–46 yrs)	- 40% reported premenstrual increase in symptoms, data confirmed by PEFRs.
Agarwal et al. (21)	- Questionnaires based data PEFRs	- 100 asthmatic women	- 23% patients had increase in symptoms with menstruation •[-]Decreased mean AM and PM PEFR values during pre or perimenstrual phase
Juniper et al. (22)	- Methacholine challenge performed longitudinally during 2 consecutive menstrual cycles	- 17 asthmatics (10 natural cycles, 7 OCP)	- No difference in FEV, medication use or methacholine challenge -Symptoms worse during menstruation
**ASTHMA and OCP USE**			
Macsali et al. (23)	- Postal questionnaire based data	- 5,791 nordic-baltic women (25–54yrs) - 961/5791 used OCP	- OCPs associated with increase risk of asthma symptoms •[-]Associations present only among normal and overweight women, not lean women.
Juniper et al. (22)	- Methacholine challenge performed longitudinally during 2 consecutive menstrual cycles	- 17 controlled asthmatics (7 on OCP)	- No difference in FEV, medication use or methacholine challenge •[-]Symptoms worse during menstruation
Nwaru et al. (24)	- Longitudinal, serial survey based data (serial Scottish Health Surveys)	- 3,257 scottish women, 16–45 yrs	- Use of hormonal contraceptive associated with increased risk of current physician diagnosed asthma (OR 0.68) and increased risk of asthma HCU (OR 0.45)
**ASTHMA AND PREGNANCY**			
Schatz et al. (25)	- Women monitored for HCU, lung function and med use before, during and after pregnancy	- 1,739 pregnant asthmatic women	- Risk of asthma exacerbation during pregnancy: severe asthmatic women > mild and moderate asthmatic women
Schatz et al. (26)	- Asthma symptom and medication diaries •[-]Spirometry during pregnancy and 3 months postpartum	- 366 pregnancies in 330 women	- During pregnancy, asthma symptoms increased in 33% women •[-]73% of these women reverted back to pre - pregnancy control by 3 months postpartum
Belanger et al. (27)	- Interview based symptom and medication data	−872 asthmatic women	- Patients who continued to use their prescribed medication had no change in asthma severity during pregnancy
Juniper et al. (28)	- Airway responsiveness, FEV1, FVC, medication use	−20 asthmatic women	- A majority of women had decreased asthma symptoms and severity during pregnancy
**ASTHMA AND MENOPAUSE**			
Gomez et al. (29)	- Postal questionnaire based data	- 2,206 nordic-baltic women 46–54 yrs - 884 OCP users and 540 HRT users	- Women taking HRT: increased risk of asthma - Women not taking HRTs: no difference in self-reported asthma between pre-menopausal and post-menopausal women
Real et al. (30)	- Questionnaire data (ECRHS II) - Lung function and hormonal serum markers measured	- 4,529 women (45–56 yrs)	- Decline in lung function and asthma symptoms: women in the menopause transition (amenorrheic for 6 months) > pre-menopausal women had lower lung function
Troisi et al. (31)	- Questionnaire data (NHS data)	- 41,202 premenopausal and 23,035 postmenopausal women	- Asthma incidence: pre-menopausal women> postmenopausal •[-]Higher incidence among postmenopausal women who had never used HRT compared to women who reported current or previous use
Triebner et al. (32)	- Questionnaire data (RHINE)	- 2,322 women aged 45–65 yrs	- A new phenotype of asthma described with onset after menopause

*AM, morning; CAMP, The childhood asthma management program; ECRHS, european community respiratory health survey; ED, emergency department; FEV, forced expiratory volume; FVC, forced vital capacity; PIAMA, prevention and incidence of asthma and mite allergy; HCU, healthcare utilization; NHS, nurses' health study; HRT, hormone replacement therapy; RHINE, respiratory health in northern europe; PEFR, peak expiratory flow rate; PM, evening; PMA, perimenstrual asthma; pub, publication; OCP, oral contraceptive pill; SABA, short acting beta agonist; SARP, severe asthma research program; yr, year*.

## Maternal Factors, Epigenetics, the Microbiome, and Asthma

Maternal factors, such as smoking, antibiotic or corticosteroid use, and prenatal stress, are associated with increased development of asthma or wheeze in children (33–36). While boys have increased development of asthma and wheeze as children, no sex specific association was determined with maternal smoking or antibiotic use and the development of wheeze in children (34, 35). Further, a prospective study showed no sex differences in maternal asthma control during pregnancy and asthma risk in offspring (37). However, previous studies showed that female infants from mothers not taking inhaled corticosteroids had reduced birth weights compared to female infants from mothers administered inhaled corticosteroids, with no change in birth weights in male infants from these groups (38). A female fetus was also associated with increased circulating monocytes compared to the male fetus in this study. A sex bias, with more boys compared to girls, was also determined with prenatal maternal stress and the development of asthma or wheeze (39). However, not all maternal exposures are adverse, as maternal exposure to farming and farm milk is protective against the development of asthma (40). Women who farmed during pregnancy and had exposure to multiple animal species and consumed farm milk led to enhanced innate immune responses through increased expression of pattern recognition receptors, upregulated IFN-γ production, upregulated T regulatory cell function and reduced Th2 cell dependent allergic inflammation in early childhood (41–43). Further, breastfeeding is known to decrease the risk of asthma in infants (44). However, sex was not addressed as a variable in some breastfeeding studies (37, 38), or was listed as a co-variate that did not impact the findings in other studies (45–49). Combined, these studies suggest that prenatal stress and potentially maternal asthma control may differentially affect male and female offspring in childhood development of asthma/wheeze.

Microbiome formation in early life is vital for educating the immune system and many environmental factors, including mode of delivery, formula feeding, antibiotic use, and exposure to animals, are important in microbiome formation and are associated with asthma. The Canadian Healthy Infant Longitudinal Development (CHILD) study determined that the microbiome composition, specifically reductions in bacterial genres *Faecalibacterium, Lachnospira, Veillonella*, and *Rothia*, at 3 months of age (but not 1 year) increased the risk of developing asthma or allergic diseases (50). No sex differences were observed in the abundance of these bacterial genres and development of asthma or allergic disease. However, additional analysis of this cohort showed a decreased abundance of *Lactobacillus* in Caucasian male infants at 3–4 months of age born to mothers' with asthma compared to female infants (51). *Lactobacillus* are associated with increased production of anti-inflammatory cytokines in cord blood and *Lactobacillus johnsonii* reduced ovalbumin or cockroach allergen-mediated airway inflammation in mouse models of asthma (52, 53).

Gene transcription can be altered by epigenetic modifications that are established *in utero* or developed due to environmental exposures, disease states, antibiotic or medication use, and lifestyle choices. Alterations in the epigenome *in utero* or during childhood may also drive asthma development (54, 55). Maternal smoking alters DNA methylation and maternal smoking is a known risk factor for childhood asthma. Further, asthmatic children have differences in DNA methylation levels at 19 different loci depending on *in utero* exposure to maternal smoking (54, 56, 57). However, none of these studies showed a sex disparity in maternal smoking and the development of asthma. Studies looking at maternal stress during pregnancy and maternal obesity have also shown similar findings, with differences in DNA methylation depending on the mother's status (54). A study investigating maternal immune status during pregnancy found that while maternal production of IL-13, IL-4, IL-5, IFN-γ, IL-10, and IL-17 during pregnancy was unrelated to childhood asthma, the ratios of IFN-γ/IL-13 and IFN-γ/IL-4 during pregnancy were associated with a decreased risk of asthma (58). *In utero* and early exposures to farm and animal barns is protective against asthma even in genetically similar Amish and Hutterite populations (59). However, no sex difference in the development of asthma was seen the Amish or Hutterite children (*n* = 30 from each population), although the sample size may not have provided the power needed to detect sex differences within and between the groups. Taken together, these studies show that maternal factors are important in the development of asthma, but it is not yet clear if there is a sex bias in the offspring developing asthma for some of these risk factors and additional studies are warranted.

## Sex Disparity in Childhood Asthma

Maternal environment and genetics are important in development of asthma during childhood. However, it is still unclear why boys have an increased prevalence of asthma compared to girls. A potential explanation is that boys have dysynaptic growth of their large airways, meaning the growth of their airway lags behind the growth of the lung parenchyma, leading to narrower airways in boys compared to girls (60). Peripheral blood mononuclear cells (PBMCs) from boys have also been shown to have increased IFN-γ in response to phytohaemagglutin stimulation compared to PMBCs from girls (61). Atopy, defined as the genetic tendency to develop allergic disease, as evidenced by specific IgE and skin prick testing to common allergens is increased in also boys compared to girls (62–64). Some studies have also shown that boys have an increased immunological response compared to girls. One study analyzing 81 infants hospitalized with a diagnosis of RSV bronchiolitis found that boys were more severely affected than girls using a severity index based on heart rate, respiratory rate, wheezing, and oxygen saturation (65). Multiple other studies found that in children, the male sex was a risk factor for acute and chronic otitis media (66–68). These different factors contribute to the sex disparity in childhood asthma and may explain the sex differences in childhood asthma.

Longitudinal studies tracking children for asthma symptoms and diagnoses of asthma through childhood, adolescence, and adulthood determined that increasing sex hormones were important for change in asthma prevalence observed in males and females. The (Prevention and Incidence of Asthma and Mite Allergy (PIAMA) cohort tracked sex differences in asthma through the first 8 years of life from questionnaires. In the PIAMA cohort, the prevalence of asthmatic wheeze, defined as wheeze that resulted in diagnosis of asthma by age 8, was increased in boys compared to girls from the first year of life. This study also showed that atopy, defined as a specific IgE > 0.70 IU/mL for at least one standard inhalant allergen (house dust mite, cat, dog, birch, grass, or mold), was more prevalent in boys than girls at age 8 years (36.4% vs. 24.0%: OR, 1.8; 95% CI, 1.3–2.5) (10). While a longitudinal study tracking early life development of wheeze and asthma, this study stopped at age 8, prior to puberty. Additional longitudinal studies have tracked asthma symptoms and prevalence from childhood through adolescence. The Childhood Asthma Management Program (CAMP) study longitudinally tracked asthma symptoms in children ages 4–18 alongside the progression of puberty, recorded by Tanner stage measurements. Prior to the start of puberty, when the Tanner stage was at 1 (ages < 7), boys had increased reporting of asthma symptoms compared to girls. However, when Tanner stages began increasing in girls, starting around age 9–10, there were also increased asthma symptoms in girls. Asthma symptoms in the boys did not increase as puberty progressed and actually declined at the end of puberty (Tanner stages 4–5) (9). Similar results were determined from the Tracking Adolescents' Individual Lives Survey (TRIALS) study. In TRIALS, the prevalence of asthma was similar in boys and girls at a mean age of 11.1. However, by age 16.3, the prevalence of asthma was significantly higher in females compared to males. This shift in the prevalence of asthma was thought to be due to an increased incidence and decreased remission of asthma in females compared to males (11). Further, in a longitudinal study, a German cohort of 274 children with current asthma at age 10 were asked about asthma and symptoms via a questionnaire at ages 14 and 20. In this cohort of children with asthma (age 10), males continued to have the higher percentage of asthma at ages 14 and 20. However, in the control group, with no asthma at age 10, females had a significant development of asthma by age 20 (6.4% of females compared to 3.3% of males) (13). Combined, these longitudinal studies suggest that during puberty, the sex associated switch in asthma prevalence may be driven by increased incidence in females.

Using large retrospective cohort data and hospitalization records, investigators were able to look at asthma care utilization during childhood and adolescence. Retrospective Kaiser-Permanent care computerized data from a cohort of 60,694 patients determined asthma care utilization and severity was higher in males compared to females from ages 2 to 13, was similar between males and females from ages 14 to 22, and was higher in females compared to males from ages 23 to 64 (12). Further, a review of 288,977 asthma related hospitalization records among the Canadian population determined that the 3 year cumulative incidence of asthma hospitalization was substantially higher for males compared to females less than 15 years of age and this pattern was reversed for adults (1). The longitudinal and retrospective studies show there is a shift in asthma prevalence and hospital utilization during puberty, but the age at which female prevalence becomes higher than male prevalence varies in the studies. This variation is likely from different patient population demographics, variations in outcome measures, and the time of sampling for each study. Nevertheless, these studies strongly suggest that the prevalence of asthma in females increases during puberty and adolescence.

The association of obesity and asthma is well-established in both pediatric and adult populations and there is increasing interest in obesity and the sex discrepancy in asthma. A study evaluating 5,984 children in Israel determined obesity was associated with increased incidence of asthma in both boys and girls. However, chest symptoms, including wheezing and shortness of breath, and asthma were reported more frequently in obese boys compared to obese girls (69). As children age, the sex and obesity associated pattern changes. A study tracking body mass index (BMI) from birth to adolescence among children with and without asthma found that BMI development differed between girls with and without asthma, with the highest BMI seen among females with persistent asthma (70). Among males in this cohort, there were no clear associations between asthma and BMI (70). Castro-Rodriguez et al. also looked at the relationship between obesity and asthma. Their study showed that females who became overweight or obese between ages 6 and 11 were seven times more likely to develop new asthma symptoms at age 11 or 13 while this pattern was not seen in females who remained lean. Conversely, males who became overweight or obese in this time frame had a similar prevalence of asthma symptoms as those who did not become overweight or obese (71). These studies showed that in the pediatric populations obesity and asthma are associated with boys, but that as adolescents the association of obesity and asthma shifts to females.

Sex-related differences in the lung development, obesity, nutrition, and responses to viral infection and environmental exposures, including farm and second-hand smoke exposure, should be considered when determining sex differences in asthma pathophysiology. While sex hormones are important in regulating the inflammatory response in asthma, other factors are also important, particularly in children prior to puberty when sex hormone levels are minimal.

## Asthma After Puberty

As discussed above, asthma incidence begins to increase in females during late adolescence and as adults women have an increased prevalence of asthma compared to men. This increased prevalence of asthma is true in the Hutterite farming population that has a similar lifestyle, except for farming practices, and asthma risk factors as the Amish, but asthma prevalence rates similar to westernized populations (59). Over a 10–13 year study period, 1,325 Hutterites between the ages of 6 and 91 (with a mean age of 26) were assessed using asthma questionnaires, pulmonary function tests, methacholine challenges and measures of atopy (72). This study found that the overall prevalence of asthma increased over the study period while prevalence of atopy stayed the same. Even further, the rise in asthma was only found among females while the prevalence among males did not change, suggesting a sex-specific response (72). Sex hormone fluctuations are frequent for women during the reproductive phase of life, and asthma symptoms are known to vary during the menstrual cycle, pregnancy, and menopause.

### Asthma and the Menstrual Cycle

Approximately 30–40% of women with asthmatic report worsening of asthma symptoms during the pre or perimenstrual phase of their menstrual cycle (14). Women with pre or perimenstrual asthma symptoms (PMA) self-reported increased use of inhaled short acting beta agonist and decreased morning peak expiratory flow rates during the perimenstrual interval of their menstrual cycle compared to women with asthma without PMA. However, there were no differences in FEV1 or methacholine-induced bronchoprovocation between the two groups (14). A study by Pauli et al. collected daily records of asthma symptoms and peak expiratory flow rates from both women with asthma and healthy controls through the follicular, mid-luteal and late luteal phases of the menstrual cycle. Within the healthy control group as well as the group of asthmatic women, there were no significant changes in spirometry and airway reactivity at any point of the menstrual cycle. However, in asthmatic women, morning peak flows and asthma symptoms (shortness of breath, cough, wheeze, and chest tightness) deteriorated significantly from the follicular to luteal phase (15). A survey conducted among women with asthma described 33% of the women had worsening pulmonary symptoms during the premenstrual period, menstrual period or both with the most significant symptom worsening noted in dyspnea, wheezing and chest tightness in the premenstrual period. Of these women whose asthma was affected by menses, 68% had noted a history of previously being hospitalized for their asthma (19). Multiple other studies have also shown premenstrual deterioration of symptoms and decrease in premenstrual peak expiratory flow rate values (20). Clinical asthma symptoms may appear after ovarian hormones have impacted inflammatory pathways, explaining why increased asthma symptoms are noted during the pre-menstrual and menstrual phases of menses when ovarian hormones levels are not at peak levels.

Data from the Severe Asthma Research Program also revealed that women with self-reported pre-menstrual worsening of asthma had increased use of oral corticosteroid bursts and increased health care utilization compared to women without PMA (16). However, studies determining emergency department utilization for asthma exacerbations during various times of the menstrual cycle are discordant. No difference in emergency department visits for asthma during the perimenstrual phase compared to other points in the menstrual cycle was determined in 792 women with acute asthma exacerbations (17). Increased emergency department visits during the preovulatory phase were determined by interviews and medical record review in 288 women with asthma (18). While the menstrual cycle clearly impacts women with asthma, the mechanisms underlying the cyclical changes and worsening of symptoms is poorly understood (73).

With variations in asthma symptoms during the menstrual cycle, investigators also evaluated changes in asthma symptoms and pulmonary function in women taking hormonal oral contraceptives. A cross sectional study using a postal survey found that contraceptive pill use in premenopausal women was associated with an increased reporting of asthma symptoms and wheezing in normal and overweight women, but not lean women (BMI < 25) (23). However, another study determined no difference in FEV1, response to methacholine, or use of asthma medications in women taking oral contraceptives compared to those with a natural menstrual cycle (22). Both the Swiss Study on Air Pollution and Lung Diseases in Adults (SAPALDIA) and data from the national Scottish Health Surveys showed similar data. The SAPALDIA study reported that women taking oral contraceptives had a decrease in airway responsiveness compared to women not on oral contraceptives and the Scottish data reported that women taking oral contraceptives had a reduced risk of physician diagnosed asthma and urgent care use (24, 74). These discordant findings suggest that additional studies are needed to determine the effects of birth control medications on asthma.

### Asthma Control Varies During Pregnancy

During pregnancy, studies have reported discordant findings in pregnancy and changing of asthma symptoms. Increased asthma symptoms, tracked by daily diaries and monthly spirometries, were reported in approximately one third of women with asthma (26). The increase in asthma symptoms was maintained until 3-month post-partum when 73% of the women had asthma symptoms revert to the pre-pregnancy course. Importantly in this study, the course of asthma was studied in successive pregnancies in 34 of these women and a statistically significant concordance in the course of asthma symptoms during the two pregnancies was found in 58.8% of the women (26). A later study showed that more severe asthma was linked to worsening of asthma symptoms during pregnancy as 12.6% of patients initially classified with mild asthma had exacerbations while pregnant, while 25.7% of patients classified as moderate and 51.9% of patients classified as severe suffered from exacberations (25). No change in asthma symptoms and asthma medication use during pregnancy was determined in symptom and medication data collected by in-person and telephone interviews from 800 women with physician diagnosed asthma during any trimester of pregnancy when patients continued to use their prescribed medications (27). Further, viral infections and non-adherence to medications were determined to be the primary triggers for asthma exacerbations during pregnancy (75). However, in a prospective study on 16 women during pregnancy showed improved airway responsiveness and asthma severity during pregnancy and a return to preconception levels 1 month after delivery (28). The National Heart, Lung, and Blood Institute and the Global Initiative for Asthma (GINA)'s current guidelines designate that during pregnancy, women should maintain their current regimen of asthma medications (76). A better understanding of how asthma symptoms will change will enable a more personalized approach for managing asthma and educating women on the importance of medication adherence during pregnancy.

### Asthma and Menopause

There are variable findings related to menopause and asthma. The European Community Respiratory Healthy Survey I (ECHRS I) analyzed questionnaire results from 2,206 women aged 46–54 years of whom 884 were menopausal and 540 had used hormone replacement therapy (HRT). This study found an increased risk of asthma in lean women taking HRT, but no differences in self-reported asthma between pre-menopausal and postmenopausal women not taking HRT (29). The ECHRS II trial then reported that women who were in the menopause transition, those who had been amenorrheic for 6 months) had lower lung function and increased asthma symptoms compared to pre-menopausal women (30). The US Nurses' Health Study data revealed that postmenopausal had a decreased incidence of asthma than pre-menopausal women (31). The magnitude of this difference was noted to be higher among the postmenopausal women who had never used HRT compared to women who reported current or previous use (31). BMI was not addressed in this study data. The etiology of the discrepancy of these studies is unclear but may be related to other associated factors including symptom reporting rates and health care seeking behavior, concordant smoking and time course of HRT initiation in relation to diagnosis of asthma. The RHINE study in addition to others have also described a new phenotype of asthma in a subset of women who have onset of the disease after menopause (32, 77). These studies illustrate that fluctuations of hormones during menopause, including the use of HRT, may contribute to asthma symptoms. However, more studies are needed to determine the extent of this effect.

### Testosterone May Be Protective Against Asthma

Given the sex disparity in asthma, male sex hormones have also been a topic of interest when studying the disease process. Using the SARP database of patients, one study looked at hormone data which was collected by blood draws assessing estradiol, progesterone, testosterone, and dehydroepiandrosterone-sulfate (DHEA-S) levels in 187 children while simultaneously analyzing Tanner stages, lung function and asthma symptom control using an asthma control questionnaire (ACQ6) (78). In males, higher DHEA-S levels were associated positively with pre and post bronchodilator FEV1% and pre-bronchodilator FVC% as well as improved ACQ6 scores while in females, higher estradiol levels were associated negatively with pre-bronchodilator FEV1% and FVC% (78). In another study evaluating 2,143 adult men, it was found that higher early morning serum testosterone and dihydrotestosterone levels were associated with a higher FEV1 and FVC (79). Our lab has shown that in humans, women with asthma had higher numbers of lung ILC2 (type 2 innate lymphoid cells) compared to men with asthma (80). Further, in adult mice, testosterone negatively affected, or lowered, the number of ILC2s showing that sex hormones do play a role in mediating inflammation in asthma (80). More studies are needed to evaluate a potential protective effect of testosterone as this would provide a better understanding of the underlying mechanism for the sex disparity that is seen in asthma.

### Obesity and Adult Asthma

As adults, obesity and asthma are associated in women but not men. The National Population Health Survey in Canada determined data from 9,149 men and women tracked longitudinally from ages 20 to 64, that a baseline BMI greater than 30 had a 1.9 odds ratio of asthma incidence compared to a baseline between BMI of 20-24.9 (normal BMI) (81). No significant association between asthma and baseline BMI was determined in men (81). Further, cross-sectional and longitudinal data from 5,114 adults found the prevalence ratio of asthma was 1.93 in obese women compared to normal weight women with no difference in men that were obese or had a normal BMI (82). A cluster analysis of adults in the Severe Asthma Research Program showed that obese women with late onset of asthma had a more severe asthma phenotype with an average forced expiratory volume in 1 s (FEV1) of 75% (a moderate reduction) and this cluster of women also required frequent oral corticosteroid use to manage exacerbations (4, 7). Similar findings, with a female predominance in clusters that had obese patients with either controlled or uncontrolled asthma, were found in a multi-variable cluster analysis with patients from the Asthma Clinical Research Network (83). Further, increased neutrophils in the sputum were also found in obese women compared to non-obese women with asthma, and no differences were determined in obese and non-obese men with asthma (84). Therefore, a sex disparity in associations with obesity and asthma was seen in cross-sectional and longitudinal studies as well as with multi-variable cluster analyses. Overall, it is unclear why obesity in women, but not men, is associated with asthma and increased asthma severity. With obesity, there are increases in adipose tissue, which is known to secrete estrogen, as well as increased leptin, an energy-regulating hormone that promotes inflammation. No differences in estrogen concentrations were determined in the serum of obese and non-obese women, (84) but increased leptin levels were detected in women compared to men, at any given measure of BMI (85). Select studies in patients with asthma undergoing bariatric surgery have also determined that reducing BMI improved asthma symptoms and FEV1 (86). However, these small studies were not powered to determine if women had a more dramatic improvement compared to men. Therefore, additional research is needed to determine the mechanisms driving the sex differences in obese asthma.

## A Sex Disparity in Other Atopic Diseases

A sex disparity is also described in other atopic conditions such as rhinitis, eczema, food allergy, vernal conjunctivitis, and drug hypersensitivity (87, 88). A systematic meta-analysis looking at 67 cross-sectional population based studies internationally found that as children (age less than 11), boys had a higher reported rate of rhinitis compared to girls (89). In this same review, in adolescents (ages between 11 and 18), significantly more females than males were affected (89). Questionnaire data amongst the Isle of Wight cohort in the UK showed that atopic rhinitis prevalence was more common in boys at age 18 and was associated with a greater positive transition in boys from age 10 to 18 (90). This study also showed that non-atopic rhinitis was greater in girls at age 18 and was associated with a greater positive transition in girls from age 10 to 18 (90). The meta-analysis did not differentiate between allergic and non-allergic rhinitis. A study in the United States and multiple studies in Europe have indicated a higher prevalence of eczema in boys vs. girls (91–93). In some studies of preschool aged children, boys were more often atopic and girls suffered significantly more from non-atopic or “intrinsic” eczema (94, 95). However, other studies showed no sex difference in prevalence of eczema (96, 97). In school aged children, there have been discordant findings with one study in Taiwan showing no sex differences in the prevalence of eczema and another from Germany showing more girls than boys suffering from eczema (98, 99). In adulthood, few studies have shown a higher female prevalence of the disease (100, 101). Similar to eczema, with food allergy, the data regarding sex is variable. A systematic literature review revealed that among children with food allergies, 64.4% were males and 35.6% were females but among adults, 34.8% were males and 64.2% were females (102). Another disease of interest in terms of sex differences is vernal conjunctivitis, a condition caused by allergies. This condition typically occurs in the younger population, between 4 and 12 years of age and more frequently amongst boys with the male to female ration ranging from 3 to 5:1. Interestingly, after puberty, the disease spontaneously disappears in majority of patients (87, 103). Sex differences have also been described in drug hypersensitivity. Overall, females have increased allergic and non-allergic drug reactions (104). A common cause of adverse drug reactions (ADRs) is anaphylaxis during anesthesia, and interestingly, there were some differences with specific medications; males were more likely to have an ADR to atracurium during surgery while females were more likely to react to suxamethonium during surgery (104). Combined, these study show a sex disparity in other atopic diseases, but a better understanding of how sex hormones regulates these diseases is warranted.

## Conclusion

Clinical studies showed a sexual dimorphism in asthma along different hormonal points of life. Prior to puberty, asthma symptoms are increased in boys compared to girls. However, after puberty, sex differences are variable during menstruation, pregnancy, and menopause. Deciphering how sex hormones regulate airway inflammation may also personalize treatment strategies for asthma-based therapeutics (including neutralizing biologics), repurpose androgens as asthma therapeutics, and determine the percentage of women and men with different asthma phenotypes are needed to test new asthma therapeutics. Further, a greater understanding of how sex hormones regulate different asthma phenotypes would enable the use (or avoidance) of hormonal therapy, help predict asthma symptoms during pregnancy, and help determine ways to control peri-menstrual and menstrual asthma (73, 105). Finally, understanding how sex hormones regulate asthma pathogenesis is crucial for treating patients during various phases of life (e.g., puberty or pregnancy), asthmatic women on hormonal birth control medications or hormone replacement therapy, or patients with comorbidities, like obesity.

## Author Contributions

RS: wrote and edited the manuscript; DN: edited the manuscript.

### Conflict of Interest Statement

The authors declare that this review article was written in the absence of any commercial or financial relationships that could be potential conflicts of interest.

## Abbreviations

ACQ: asthma control questionnaire
ADR: adverse drug reactions
BAL: bronchoalveolar lavage fluid
BMI: body mass index
CAMP: childhood asthma management program
CHILD: canadian healthy infant longitudinal development
DHEA-S: dehydroepiandrosterone-sulfate
ECHRS: european community respiratory healthy survey
FEV1: forced expiratory volume in 1 s
GINA: global initiative for asthma
HRT: hormone replacement therapy
IFN: interferon
IL: interleukin
ILC2: group 2 innate lymphoid cells
PBMCs: Peripheral blood mononuclear cells
PIAMA: prevention and incidence of asthma and mite allergy
PMA: pre or perimenstrual asthma symptoms
SAPALDIA: swiss study on air pollution and lung diseases in adults
TRAILS: tracking adolescents' individual lives survey.
